# Catalytic Oxidations in a Bio-Based Economy

**DOI:** 10.3389/fchem.2020.00132

**Published:** 2020-02-28

**Authors:** Roger A. Sheldon

**Affiliations:** ^1^School of Chemistry, Molecular Sciences Institute, University of the Witwatersrand, Johannesburg, South Africa; ^2^Department of Biotechnology, Delft University of Technology, Delft, Netherlands

**Keywords:** bio-based economy, biomass, biocatalysis, catalytic oxidation, alcohol oxidases, carbohydrates, waste valorization

## Abstract

The role of bio- and chemo-catalytic aerobic oxidations in the production of commodity chemicals in a bio-refinery is reviewed. The situation is fundamentally different to that in a petrochemicals refinery where the feedstocks are gaseous or liquid hydrocarbons that are oxidized at elevated temperatures in the vapor or liquid phase under solvent-free conditions. In contrast, the feedstocks in a biorefinery are carbohydrates that are water soluble solids and their conversion will largely involve aerobic oxidations of hydroxyl functional groups in water as the solvent under relatively mild conditions of temperature and pressure. This will require the development and use of cost-effective and environmentally attractive processes using both chemo- and biocatalytic methods for alcohols and polyols.

## Introduction

One of the grand challenges of the twenty-first century is the implementation of the transition from an unsustainable economy based on fossil resources–oil, coal, and natural gas—to a sustainable, carbon-neutral economy based on the use of renewable biomass. This switch to a so-called bio-based economy is urgently required in order to mitigate global warming caused by increasing carbon dioxide emissions to the atmosphere. First generation (1G) renewable raw materials, exemplified by corn starch, sugar cane, and sugar beet, are not perceived as sustainable options in the long term as their utilization involves, directly, or indirectly, competition with food production. In contrast, the use of second generation (2G) renewable biomass, in the form of waste polysaccharides, such as lignocellulose (Liguori and Faraco, 2016; Zhang et al., 2017) and pectin, from agricultural and forestry residues and food supply chain waste (Dahiya et al., 2018), is perceived as a sustainable long term option for producing biofuels and commodity chemicals (Sheldon, 2014, 2016, 2018; Horváth et al., 2017). Looking further afield, third generation (3G) aquatic biomass, such as micro- and macro-algae and cyanobacteria, has additional advantages (John et al., 2011; Al Abdallah et al., 2016; Shuba and Kifle, 2018). For example, there is no requirement for arable land and fresh water for their production and they have much higher growth rates than terrestrial plants. On the other hand, there are substantial technical problems associated with their production and conversion which, in the short term, represent a significant hurdle to be overcome for commercial viability.

## Carbohydrates to Commodity Chemicals in a Biorefinery

**Figure 1:**
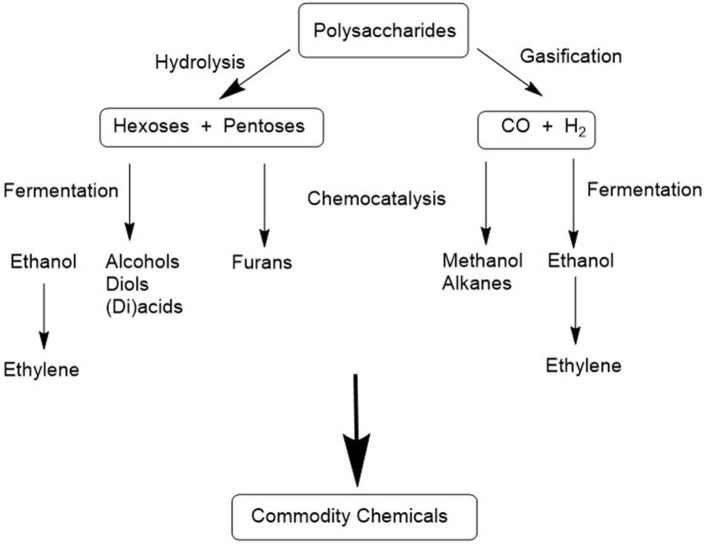
Conversion of polysaccharides to biofuels and commodity chemicals in a biorefinery.

In a petrochemical refinery the basic chemicals are lower olefins (ethylene, propylene, and butenes) and aromatics (BTX: benzene, toluene, and xylenes), together with carbon monoxide and hydrogen (syn gas). The hydrocarbons are gases or hydrophobic liquids. They are converted with petrochemical catalytic technologies, particularly catalytic oxidation with dioxygen, to a variety of commodity chemicals, usually in solvent-free systems. In contrast, the basic chemicals in a bio-refinery will be C_6_ and C_5_ sugars produced by hydrolysis of polysaccharide feedstocks, and/or syn gas produced by their gasification. The carbohydrates are hydrophilic, water soluble solids. Several scenarios can be envisaged for further conversion to commodity chemicals (Figure 1):

- Syn gas could be converted to commodity chemicals by applying existing catalytic technologies used in petrochemical refineries or by fermentation (Phillips et al., 2017; Asimakopoulos et al., 2018).
- Monosaccharides such as glucose could be converted to a variety of lower alcohols, diols, carboxylic acids, and dicarboxylic acids by fermentation. Indeed, fermentation is already the commercially most viable route to many of these products with lactic acid, 1,3-propane diol and 1,4-butane diol as prominent examples.
- Bioethanol, produced as a carbon neutral fuel, could be converted to ethylene and a variety of other products using established technologies (see Figure 1), including catalytic aerobic oxidations.
- Chemo- or bio-catalytic conversion of monosaccharides, such as glucose, to commodity chemicals will require in many cases removal of oxygen, by hydrogenolysis and/or dehydration. Catalytic oxidations in a biorefinery involve oxidation of alcoholic OH groups in monosaccharides, or even the polysaccharide precursors, or their downstream products and further oxidation of the resulting carbonyl compounds.

## Catalytic Oxidations

A key reaction in organic synthesis is the oxidation of primary and secondary alcohols to give the corresponding aldehydes or carboxylic acids and ketones, respectively. Traditionally these transformations were performed with stoichiometric quantities of inorganic oxidants, notably chromium (VI) compounds such as the Jones reagent (CrO_3_ and sulfuric acid). However, such procedures are not atom efficient and lead to the formation of copious amounts of toxic, chromium-containing waste, i.e., high E factors and problematic waste disposal issues. Consequently, in the last two decades such methods have been increasingly replaced by atom efficient catalytic alternatives involving dioxygen or hydrogen peroxide as the terminal oxidant.

Interestingly, the history of catalytic oxidations of carbohydrates (Arts et al., 1997) predates the oxidations of lower olefins and aromatics that form the basis of the petrochemical industry. The aerobic oxidation of mannose over a platinum black catalyst, for example, dates from 1861 (von Gorup-Besanez, 1861) and many supported noble metal catalyzed aerobic oxidations of carbohydrates were developed in the first half of the last century. At the turn of the century, we developed an aqueous biphasic system for the aerobic oxidation of primary and secondary alcohols to the corresponding aldehydes and ketones, respectively, in a solvent free system using a water-soluble palladium complex of bathophenanthroline (ten Brink et al., 2000). This system could also be effective in the aerobic oxidation of water soluble alcohols, including carbohydrates. Indeed, there are many examples of the aerobic oxidation of alcohols catalyzed by precious metals such as palladium, platinum and gold (Stahl, 2004; Parmaggiani and Cardona, 2012). However, in the context of the conversion of carbohydrates to large volume, low-priced commodity chemicals, precious metals such as palladium have the disadvantage that the future availability of these scarce, “endangered elements” at cost-effective prices is rather unpredictable. Indeed, in contrast to most materials, their price tends to increase with increasing usage. Another disadvantage of noble metal catalyzed oxidations is their functional group intolerance. First row, more earth abundant metals tend to be more functional group tolerant.

**Figure 2:**
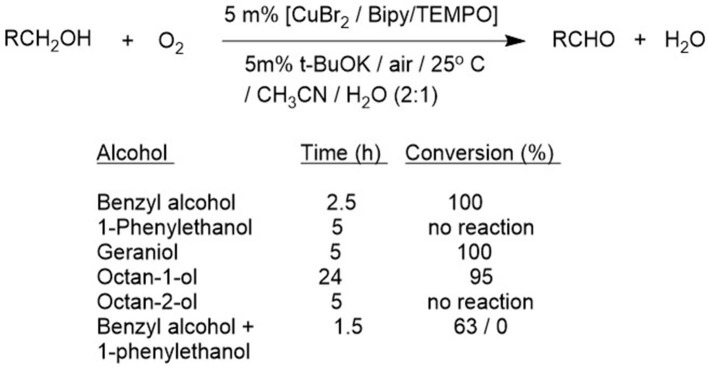
Aerobic oxidation of primary alcohols catalyzed by Cu/TEMPO.

Consequently, alternative methods have been developed that use “earth abundant” metals, such as copper and iron, as catalysts. One method with broad applications in the selective oxidation of primary alcohols to aldehydes, even in the presence of secondary alcohols, involves the combination of a Cu(II)-bipyridine (Cu-bpy) complex with a base, such as potassium hydroxide, a stable nitroxyl radical, exemplified by 2,2,6,6-tetramethyl-1-piperidine-N-oxyl (TEMPO) and its derivatives, at ambient temperature with air in aqueous acetonitrile (Figure 2; Gamez et al., 2003; Sheldon and Arends, 2004; Marais and Swarts, 2019). The generally accepted mechanism involves as the key, rate determining step, abstraction of a hydrogen atom from an alkoxide ligand by a coordinated nitroxyl radical analogous to that involved in the aerobic oxidation of primary alcohols catalyzed by the copper-dependent oxidase, galactose oxidase (Dijksman et al., 2003). An improved procedure, using Cu(I) salts with TEMPO and bipy in combination with N-methylimidazole as a base in acetonitrile as solvent was subsequently described by Stahl and coworkers (Hoover and Stahl, 2011). Furthermore, extensive mechanistic studies confirmed the copper-centered galactose oxidase-like mechanism for these systems (Geiβlmeir et al., 2005; Hoover et al., 2013).

**Figure 3:**
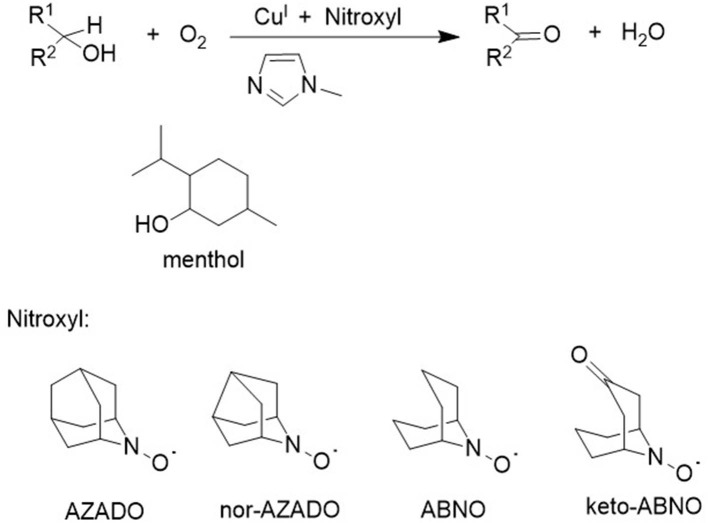
Catalytic aerobic oxidation of secondary alcohols with Cu (I)/nitroxyl catalysts.

The lack of reactivity of secondary alcohols was attributed to steric hindrance in the abstraction of an α-hydrogen atom from a coordinated alkoxide by a coordinated TEMPO ligand. Consequently, the use of sterically less hindered nitroxyl radicals such as AZADO and ABNO, respectively, in combination with Cu(I) complexes, were developed by the groups of Iwabuchi (Shibuya et al., 2006, 2011; Iwabuchi, 2013) and Stahl (Steves and Stahl, 2013), for the aerobic oxidation of secondary alcohols (Figure 3), including sterically demanding alcohols such as menthol and a variety of unprotected amino alcohols (Sasano et al., 2014).

**Figure 4:**
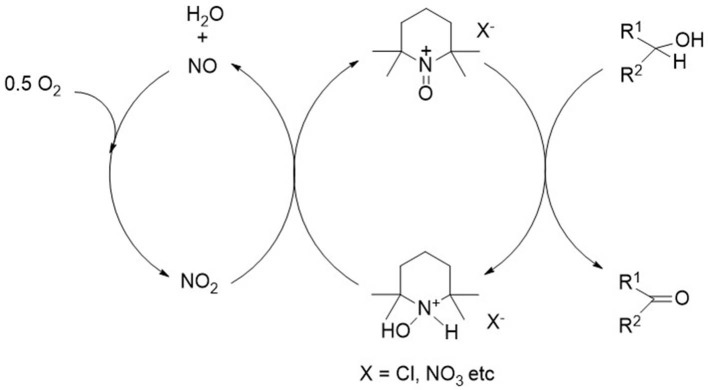
Nitric acid catalyzed aerobic oxidation of alcohols.

More recently, Ma and coworkers reported the use of an Fe(III)/4-hydroxyTEMPO/NaCl combination as a catalyst for the aerobic oxidation of both primary and secondary alcohols (Jiang et al., 2016, 2019). Other metals, including manganese, cobalt and vanadium, have also been used in combination with nitroxyl radicals (Cao et al., 2014) Transition metal free nitroxyl systems have also been described, usually involving nitrogen dioxide as the active co-catalyst. The commercially most attractive source of the NO_2_ cocatalyst is nitric acid (Kuang et al., 2010). For example, nitric acid or NaNO_2_ or a mixture of both was used, in combination with ABNO or keto-ABNO, for the selective aerobic oxidation of secondary alcohols (Figure 4; Lauber and Stahl, 2013). Interestingly, the combination of nitric acid and NaNO_2_ catalyzes the aerobic oxidation of alcohols even in the absence of a stable nitroxyl radical. The reaction involves an alkyl nitrite intermediate which decomposes to the carbonyl compound and HNO which is reoxidized by dioxygen (Aellig et al., 2011). Unfortunately, the greenhouse gas, nitrous oxide (N_2_O), can be irreversibly formed as a byproduct.

## Catalytic Oxidations in a Bio-based Economy

Although these various nitroxyl radical-based catalysts have been widely used in the aerobic oxidations of alcohols they have generally involved relatively simple primary and secondary alcohols in organic solvents, sometimes mixed with water. This was done with the development of green syntheses of, for example, active pharmaceutical ingredients (APIs) and flavors and fragrances, in mind. However, in a bio-based economy it is of interest to use these methodologies in the selective oxidation of renewable carbohydrates or key alcohols, diols, and polyols derived from them, and this involves in many cases aqueous solutions of solid substrates.

Water has both advantages and limitations as a solvent for aerobic oxidations. For example, oxidations with oxygen are much safer as there is no formation of explosive mixtures of oxygen with volatile organic solvents in the gas phase. In a typical process oxygen is supplied by bubbling air through the solution. However, the transfer of oxygen from the gas to the liquid phase is notoriously slow owing to its low solubility in water under typical operating conditions (0.268 mM at 25°C and 1 bar air) which limits the maximum space time yield to 200 mmol/L/h (Pedersen et al., 2015). This problem was alleviated in the Cu/TEMPO system by using air-microbubble techniques to facilitate gas absorption into the liquid phase (Mase et al., 2011). Alternatively, the rates of enzymatic aerobic oxidations were increased by a factor of 100 in continuous flow operation compared to the conventional batch operation (Chapman et al., 2018; Hone and Kappe, 2019). Another disadvantage of water as a solvent is that its relatively high heat capacity, compared to volatile organic solvents, translates to high energy costs for its removal by distillation.

**Figure 5:**
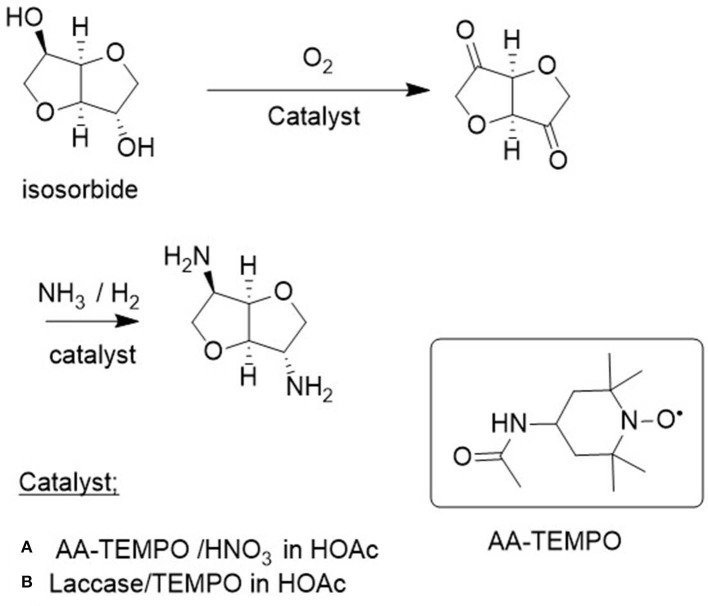
Aerobic oxidation of isosorbide to the corresponding diketone. **(A)** AA-TEMPO/HNO_3_ in HOAc. **(B)** Laccase/Tempo in HOAc.

A pertinent example, from the viewpoint of the bio-based economy, is the use of acetylamino-TEMPO (AA-TEMPO) together with nitric acid as the cocatalyst for the aerobic oxidation of primary and secondary alcohols to the corresponding aldehydes and ketones, respectively, in acetic acid or water as the solvent (Dingerdissen et al., 2011). The method was particularly useful for the oxidation of the key biomass-derived diol, isosorbide, to the corresponding diketone (Figure 5; Klasovsky et al., 2015). This is particularly surprising because of the low reactivity of the shielded *endo* OH group in isosorbide.

**Figure 6:**
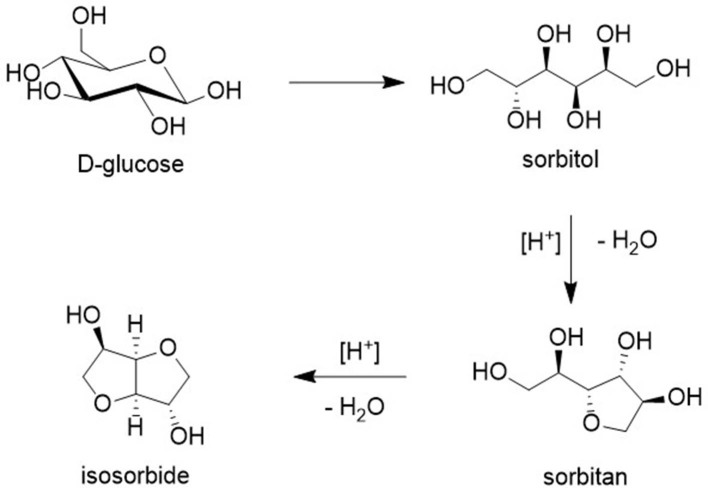
Isosorbide as a platform chemical.

Isosorbide is a commercially interesting platform chemical produced by hydrogenation of glucose to sorbitol followed by dehydration (Figure 6). It has interesting features as an industrial monomer based on its rigidity, chirality, and non-toxicity (Fenouillot et al., 2010). For example, reaction with dicarboxylic acids (or esters) affords polyesters. Alternatively, oxidation to the corresponding diketones, followed by reductive amination, affords the corresponding bis-primary amine (Figure 5) which can be converted to polyamides by reaction with dicarboxylic acids (esters).

## Biocatalysis *in aqua*: The Natural Solution

As was noted elsewhere, biocatalysis is green and sustainable (Hollmann et al., 2011; Sheldon and Woodley, 2018), conforming to 10 of the 12 principles of green chemistry, and the catalyst is non-toxic, biocompatible and biodegradable. Moreover, enzymatic reactions are generally performed in water, a particularly suitable medium for conversions of polysaccharides in a bio-based economy. Furthermore, carbohydrates tend to be ideal substrates and generally have a stabilizing effect on enzymes. In contrast with precious metal catalysts, the long term availability and price stability of enzymes is assured since they are produced from inexpensive, readily available biomass. Moreover, industrial scale oxidations employing precious metal catalysts often involve a costly purification step to remove traces of the metal in the product. In contrast, no costly removal of trace amounts of enzymes are needed in enzymatic oxidations and any enzyme ending up in aqueous effluent undergoes facile biodegradation.

### Laccase/Nitroxyl Radical Combinations

Laccases (EC 1.10. 3.2) are a diverse group of extracellular, copper-dependent oxidases. They are produced, for example, by white rot fungi, and play a key role in the delignification of lignocellulose *in vivo* (Rochefort et al., 2004). There is considerable commercial interest in the use of laccases in the pulp and paper industry and in waste water remediation in general (Gasser et al., 2014; Singh et al., 2018; Unuofin et al., 2019). They have broad substrate specificity and use dioxygen to oxidize a wide variety of, *inter alia*, phenols and aromatic amines *in vivo*. In combination with so-called mediators, notably TEMPO, they are able oxidize alcohols as was first shown by Fabbrini et al. (2001). These reactions involve one-electron oxidation of the TEMPO by the laccase to give the oxoammonium cation which is the active oxidant (Arends et al., 2006a). The reduced form of laccase is then reoxidized by dioxygen.

The laccase/TEMPO system was shown to catalyze the aerobic oxidation of primary and secondary aliphatic alcohols and 10 mol% was sufficient to give good conversions and excellent selectivities (Arends et al., 2006b). Interestingly, Ying and coworkers (Zhu et al., 2014) obtained superior results with 5 mol% laccase/AZADO, especially in the aerobic oxidation of complex and highly functionalized alcohols. Suicide inactivation is a problem with laccases since at high substrate conversions the oxoammonium cation can oxidize reactive groups in the protein or in the associated glycosyl moieties on the periphery of the enzyme (laccases are glycosylated enzymes). The stability of laccases under the reaction conditions can be significantly increased by immobilization as cross-linked enzyme aggregates (CLEAs) (Matijosyte et al., 2010).

**Figure 7:**
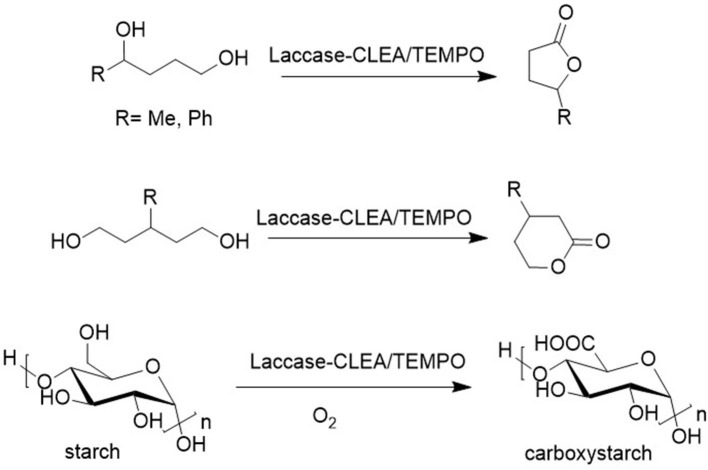
Laccase/TEMPO catalyzed oxidation of diols and polyols.

The laccase/TEMPO system also catalyzed the aerobic oxidation of the renewable diol, isosorbide to the corresponding diketone (see section Catalytic Oxidations and Figure 5B) in >99% yield (Gross et al., 2014). Similarly, 1,4 and 1,5-diols were oxidized to the corresponding lactones (Figure 7, Diaz-Rodriguez et al., 2012) and immobilization of the laccase as cross-linked enzyme aggregates (CLEAs) enabled multiple recycling (Sheldon et al., 2013).

### Copper and Flavin Dependent Alcohol Oxidases

**Figure 8:**
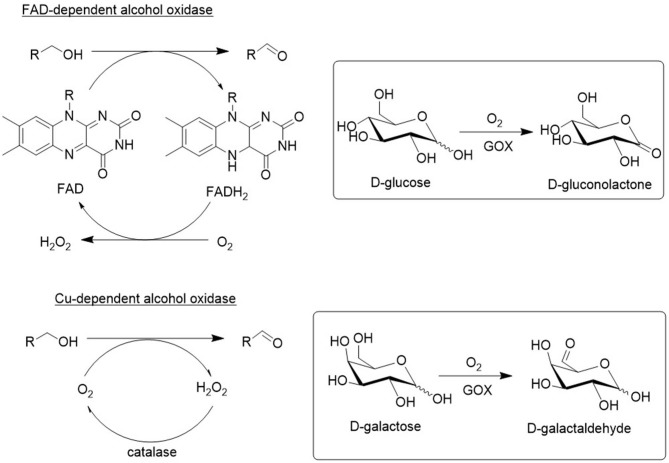
Types of alcohol oxidases.

As shown in Figure 8, there are two types of alcohol oxidases: Cu-dependent and flavin adenine dinucleotide (FAD). Both generate an equivalent of hydrogen peroxide as the coproduct and catalase is added to decompose it back to oxygen and water. Alternatively, catalase can be used to generate oxygen *in situ*. A major shortcoming of wild-type oxidases is their substrate specificity. For example, galactose oxidase (GOase) and glucose oxidase (GOX) and are very specific for galactose and glucose, respectively. Indeed, these enzymes have evolved *in vivo* to be very efficient in converting their natural substrate. However, in order to be useful in organic synthesis they need to be active and selective with a variety of alcohol substrates, particularly highly functionalized alcohols. Fortunately, this can be achieved with protein engineering using *in vitro* evolution.

**Figure 9:**
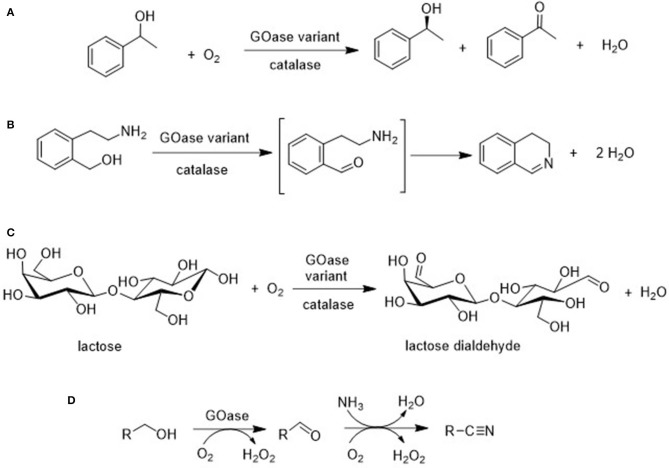
Biocatalytic aerobic oxidation of **(A)** 1-phenylethanol, **(B)** an amino alcohol, **(C)** lactose, and **(D)** ammoxidation of a primary alcohol.

For example, Turner and coworkers used directed evolution techniques to produce GOase variants that catalyze the oxidation of secondary alcohols (Figure 9A; Escaletters and Turner, 2008) and amino alcohols (Herter et al., 2015; Figure 9B). Similarly, a GOase variant catalyzed the aerobic oxidation of lactose, a disaccharide formed as a waste stream (whey) in cheese manufacture, to form the dialdehyde (Figure 9C; Cosgrove et al., 2019). The latter is of interest as a raw material for polymers. Interestingly, a GOase variant was also shown to catalyze the synthesis of nitriles by ammoxidation of primary alcohols (Figure 9D; Vilim et al., 2018).

**Figure 10:**
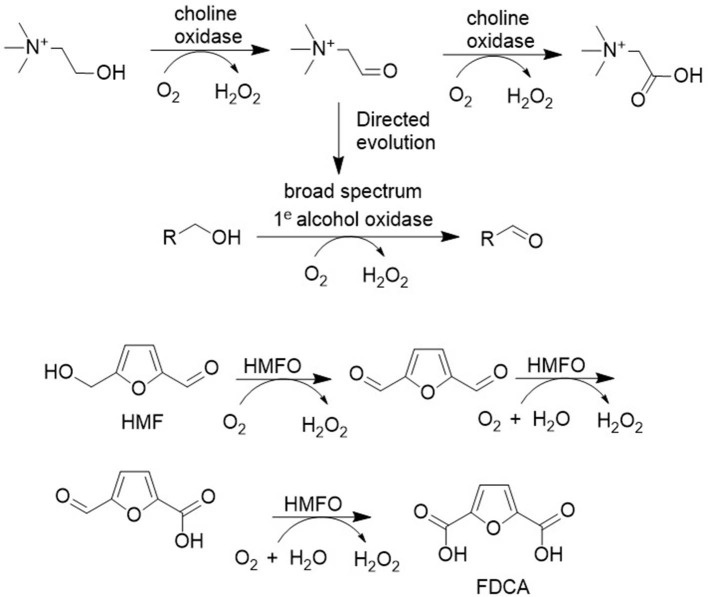
Aerobic oxidations catalyzed by FAD-dependent alcohol oxidase variants.

Structure directed evolution was also used to develop variants of the FAD-dependent choline oxidase that catalyze the aerobic oxidation of a broad range of primary alcohols to the corresponding aldehydes (Figure 10; Heath et al., 2019). Similarly, FAD-dependent HMF oxidase was engineered to effectively catalyze all three oxidation steps in the conversion of HMF to FDCA (see later).

**Figure 11:**
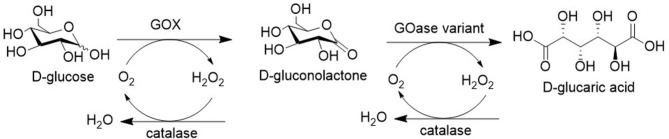
Hypothetical biocatalytic oxidation of glucose to glucaric acid.

Another reaction of industrial interest is the aerobic oxidation of glucose to glucaric acid. A hypothetical process involving two steps with a mixture of GOX and a GOase variant which is able to accept gluconolactone as a substrate is shown in Figure 11. Alternatively, it can be produced by aerobic oxidation of glucuronic acid, a building block derived from (waste) pectin (see section Acid-Catalyzed Dehydration of Carbohydrates to Furan Derivatives). Glucaric acid is of interest as an industrial monomer in itself (Wu et al., 2019) and can also be hydrogenated to adipic acid, the raw material for Nylon 6.

### Alcohol Dehydrogenases

**Figure 12:**
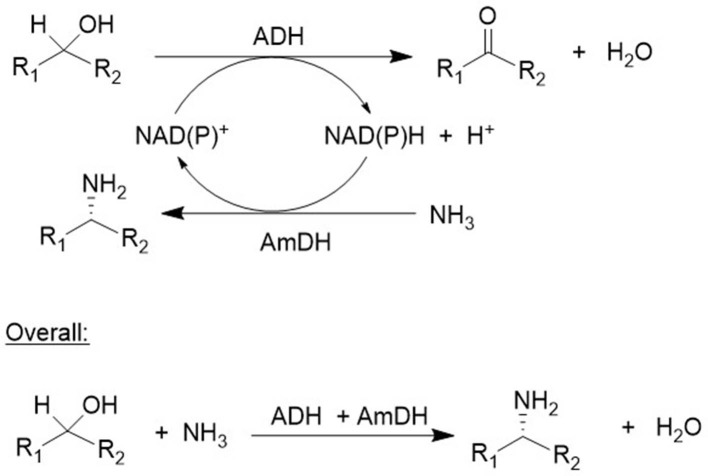
Enzymatic conversion of an alcohol to an amine.

Alcohol dehydrogenases (ADHs) catalyze the oxidation of alcohols by utilizing a nicotinamide cofactor which has to be regenerated *in situ* using an excess of a co-substrate (Kroutil et al., 2004a; Weckbecker et al., 2010). Alternatively, NAD(P)H oxidase (NOx) can be employed to catalyze reoxidation of the cofactor by oxygen (Kroutil et al., 2004b; Zhang et al., 2016). Yet another possibility is to couple the oxidation step with a reduction step, to afford an overall redox neutral process by employing so-called hydrogen borrowing, a concept which itself was borrowed from chemocatalysis literature (Hamid et al., 2007). For example, combination of an ADH with an amine dehydrogenase (AmDH) affords a redox-neutral conversion of a racemic alcohol to a single enantiomer of the corresponding amine (Figure 12; Mutti et al., 2015). Ironically, it requires the use of an aselective ADH (Thompson and Turner, 2017) because it has to catalyze the oxidation of both alcohol enantiomers, which is not a simple task as most ADHs are highly enantioselective. The overall efficiency of the process, which constitutes a conversion of an OH to an NH_2_ group, was improved by co-immobilization of the ADH and AmDH (Böhmer et al., 2018). When the alcohol, or polyol, is readily available this would be an industrially attractive way to produce the corresponding (poly)amine. A pertinent example is the conversion of isosorbide to the diamine discussed in section Catalytic Oxidations.

### Direct Oxidation of Polysaccharides

It is also of commercial interest to oxidize polysaccharides, e.g., starch and cellulose, directly to the corresponding polycarboxylic acids. Carboxystarch, for example, has potential applications as a biodegradable water super absorbent. Polysaccharides can be readily oxidized using NaOCl as the stoichiometric oxidant and TEMPO or derivatives as the catalyst (Ponedel'kina et al., 2010). However, for a commercially and environmentally attractive process it should preferably use oxygen as the stoichiometric oxidant. The laccase/TEMPO system (see above) catalyzes the aerobic oxidation of the primary alcohol moieties in starch affording carboxystarch (Viikari et al., 1999) but the relatively high enzyme costs, owing to its instability under the oxidizing reaction conditions, form an obstacle to commercialization. The stability was improved by immobilization as a cross-linked enzyme aggregate (CLEA) (Matijosyte et al., 2010).

## Acid-catalyzed Dehydration of Carbohydrates to Furan Derivatives

**Figure 13:**
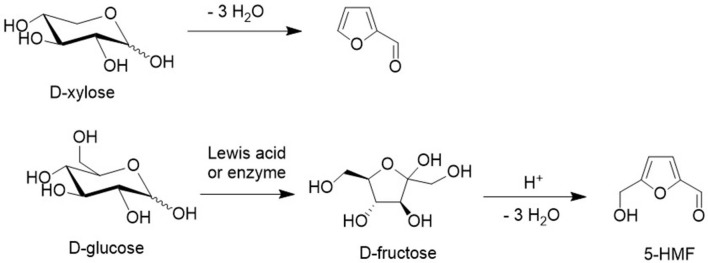
Acid catalyzed dehydration of xylose and glucose.

Acid catalyzed dehydration of pentoses and hexoses produces furfural and 5-hydroxymethylfurfural (HMF) (Tong et al., 2010), respectively. Furfural is an important commodity chemical (Lange et al., 2012) and HMF has the potential to become one (van Putten et al., 2013; Kucherov et al., 2018). Initial isomerization of D-glucose to D-fructose is followed by acid catalyzed dehydration (Figure 13) but in yields that are not conducive to commercial viability owing to the limited stability of HMF in the acidic reaction medium (Wang et al., 2017). However, according to a recent report HMF can be obtained in 95% yield by conducting the reaction with D-fructose under continuous flow conditions (Galaverna et al., 2018).

**Figure 14:**
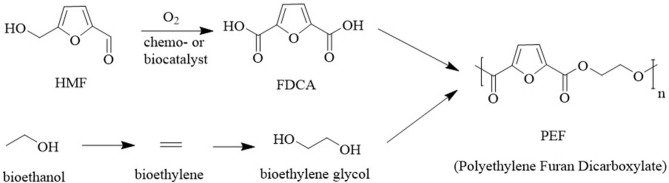
Catalytic aerobic oxidation of HMF to FDCA.

Polyethylene furandicarboxylate (PEF), which was developed by Avantium^1^ is produced from furan-2,5-dicarboxylic acid (FDCA) and ethylene glycol It is seen as a renewable alternative for fossil-based polyethylene terephthalate (PET). In addition to reducing CO_2_ emissions, PEF has superior mechanical, thermal, and gas barrier properties to PET. The key raw material, FDCA, can be produced in excellent yields by aerobic oxidation of HMF using supported precious metal catalysts (Liu et al., 2015; Zhang and Deng, 2015; Zheng et al., 2017; Motagamwala et al., 2018) or an engineered flavin-dependent alcohol oxidase (Dijkman et al., 2015) or whole cell biocatalysts (Koopman et al., 2010) in aqueous media (Figure 14).

**Figure 15:**
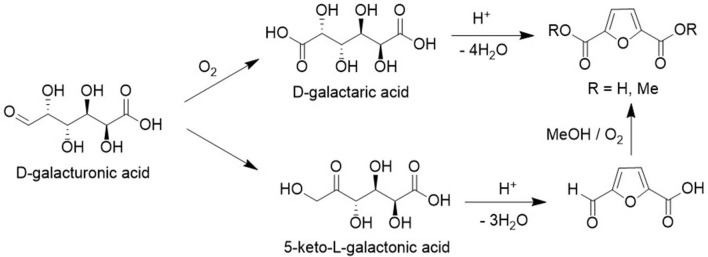
Alternative routes to FDCA.

FDCA can also be produced from uronic acids present in various agricultural residues. D-galacturonic acid, for example, is available in large quantities from the pectin in sugar beet pulp (Leijdeckers et al., 2013) and D-glucuronic acid is one of the main constituents of pectin in certain soft- and hardwoods. Aerobic oxidation of uronic acids over gold catalysts (van Es et al., 2013) affords the corresponding aldaric acids that can subsequently be dehydrated to FDCA (Figure 15; Miller et al., 2017). Alternatively, uronic acids can be isomerized to the corresponding 5-keto aldonic acids which can be converted to FDCA dimethyl ester by acid catalyzed cyclodehydration to the methyl ester of 5-formyl-2-furoic acid in methanol followed by Au/C catalyzed aerobic oxidation (van der Klis et al., 2017).

Finally, we note that this review is focused on the conversion of the carbohydrate fractions of feedstocks to commodity chemicals in a biorefinery. In practice, for commercial viability both the carbohydrate and the lignin fractions will be converted to both commodity chemicals and biofuels and this will involve both chemo- and biocatalytic methods in water (Rinaldi et al., 2016; Bugg et al., 2019).

## Conclusions and Prospects

Remarkable progress has been made in the last two decades in the development of green and sustainable catalytic methodologies for the aerobic oxidations of primary and secondary alcohols to aldehydes and ketones, respectively. However, a cursory perusal of the literature reveals that we have hardly scratched the surface with regard to the application of such catalytic methodologies to the valorization of bio-based feedstocks and key platform chemicals in biorefineries. Recent developments in the engineering of oxidative enzymes, such as copper- and flavin-dependent alcohol oxidases, using directed evolution techniques, strongly suggest that industrially viable methods for catalytic oxidations relevant to a bio-based economy will be forthcoming in the near future.

One could say that glucose is the new ethylene, and possibly propylene and butenes all rolled into one. In the words of Primo Levi: “It is the destiny of wine to be drunk and it is the destiny of glucose to be oxidized.”

## Author Contributions

The author confirms being the sole contributor of this work and has approved it for publication.

### Conflict of Interest

The author declares that the research was conducted in the absence of any commercial or financial relationships that could be construed as a potential conflict of interest.

## Footnotes

^1^https://www.avantium.com/blog/pef-the-polymer-for-the-future/
